# Sex-Specific Obesity and Cardiometabolic Disease Risks in Low- and Middle-Income Countries: A Meta-Analysis Involving 3 916 276 Individuals

**DOI:** 10.1210/clinem/dgad599

**Published:** 2023-11-01

**Authors:** Thaís Rocha, Eka Melson, Javier Zamora, Borja Manuel Fernandez-Felix, Wiebke Arlt, Shakila Thangaratinam

**Affiliations:** Institute of Metabolism and Systems Research (IMSR), University of Birmingham, Birmingham B15 2TT, UK; Department of Endocrinology and Diabetes, University Hospitals Birmingham NHS Foundation Trust, Birmingham B15 2GW, UK; Institute of Metabolism and Systems Research (IMSR), University of Birmingham, Birmingham B15 2TT, UK; Department of Endocrinology and Diabetes, University Hospitals Birmingham NHS Foundation Trust, Birmingham B15 2GW, UK; Institute of Metabolism and Systems Research (IMSR), University of Birmingham, Birmingham B15 2TT, UK; Clinical Biostatistics Unit, Hospital Universitario Ramón y Cajal de Investigación Sanitaria (IRYCIS), CIBER de Epidemiología y Salud Pública, Instituto de Salud Carlos III, Madrid 28034, Spain; WHO Collaborating Centre for Global Women's Health, Institute of Metabolism and Systems Research (IMSR), University of Birmingham, Birmingham B15 2TT, UK; Clinical Biostatistics Unit, Hospital Universitario Ramón y Cajal de Investigación Sanitaria (IRYCIS), CIBER de Epidemiología y Salud Pública, Instituto de Salud Carlos III, Madrid 28034, Spain; Institute of Metabolism and Systems Research (IMSR), University of Birmingham, Birmingham B15 2TT, UK; NIHR Birmingham Biomedical Research Centre, University Hospitals Birmingham NHS Foundation Trust and University of Birmingham, Birmingham B15 2TQ, UK; Medical Research Council London Institute of Medical Sciences (MRC LMS), London W12 0HS, UK; Institute of Metabolism and Systems Research (IMSR), University of Birmingham, Birmingham B15 2TT, UK; WHO Collaborating Centre for Global Women's Health, Institute of Metabolism and Systems Research (IMSR), University of Birmingham, Birmingham B15 2TT, UK; NIHR Birmingham Biomedical Research Centre, University Hospitals Birmingham NHS Foundation Trust and University of Birmingham, Birmingham B15 2TQ, UK; Birmingham Women's and Children's NHS Foundation Trust, Birmingham B15 2TG, UK

**Keywords:** women's health, obesity, developing countries, meta-analysis, sex-specific

## Abstract

**Context:**

There is limited knowledge about the disparities between the sexes in obesity prevalence and associated cardiovascular complications in low- and middle-income countries (LMICs).

**Objective:**

We undertook a systematic review and meta-analysis to assess sex-specific disparities in the prevalence of obesity and cardiometabolic diseases in LMICs, the burden in women, and variations by region, country's income status, setting, and time.

**Methods:**

We searched major databases from inception to March 2023. Two independent reviewers selected the studies, assessed their quality, and extracted data. We used DerSimonian and Laird random-effects models to obtain pooled estimates of odds ratios and 95% CI for the association between sex and obesity and cardiometabolic diseases, and multilevel random-effects logistic regression models to estimate the prevalence of relevant outcomes (PROSPERO CRD42019132609).

**Results:**

We included 345 studies (3 916 276 individuals). The odds of obesity were 2.72-fold higher in women than men (OR 2.72; 95% CI, 2.54-2.91). The sex-specific disparities varied by region, with the greatest disparities in Sub-Saharan Africa (OR 3.91; 95% CI, 3.49-4.39). Among women in LMICs, 23% (95% CI, 21%-25%) had obesity, 27% (95% CI, 24%-29%) had hypertension, and 7% (95% CI, 6%-9%) had type 2 diabetes. The prevalence of obesity and type 2 diabetes in women varied by region, country's income, and setting, with the highest prevalence in the Middle East and North Africa, upper-middle-income countries and urban settings. The odds of hypertension (OR 2.41; 95% CI, 1.89-3.08) and type 2 diabetes (OR 2.65; 95% CI, 1.76-3.98) were doubled in women with vs without obesity.

**Conclusion:**

There is an urgent need for a women-centred and region-stratified approach to tackle obesity awareness, treatment, and prevention in women in LMICs.

Obesity is a major risk factor for severe morbidity and all-cause mortality globally. It is associated with cardiometabolic diseases such as hypertension, type 2 diabetes mellitus, dyslipidemia, and coronary heart disease (1, 2). In women, excess body weight is also associated with sex-specific conditions, such as polycystic ovary syndrome, gestational diabetes, and hypertensive disorders of pregnancy (3-5). Notably, 6 in 10 individuals with obesity live in low- and middle-income countries (LMICs), which are facing an epidemiological transition from infectious to noncommunicable diseases (6-9).

Obesity and its complications may affect women and men differently (10). The Noncommunicable Diseases Risk Factor Collaboration (NCD-RisC) reported the burden of both underweight and obesity to be higher in women than men globally (8). Accurate data on obesity and cardiovascular disease burden is the first critical step toward reducing the global burden of cardiovascular disease in women by 2030 (11). However, the extent of sex-related differences in the risk of obesity and cardiometabolic diseases in LMICs and the variations in disparities between regions, country's income status, setting, and over time is not known. Individual studies focus on particular conditions or regions, often reporting imprecise estimates. There is also a need to assess the strength of the association between obesity and cardiometabolic diseases in women in LMICs (12).

We undertook a systematic review and meta-analysis to determine the magnitude of sex-specific differences in the prevalence of obesity and cardiometabolic diseases in LMICs, their burden on women, and if these estimates vary by World Bank region, country’s income status, setting, and over time. We also assessed the prevalence of cardiometabolic diseases, like hypertension and type 2 diabetes, in women with vs without obesity in LMICs.

## Methods

### Search Strategy and Selection Criteria

**Figure 1.**
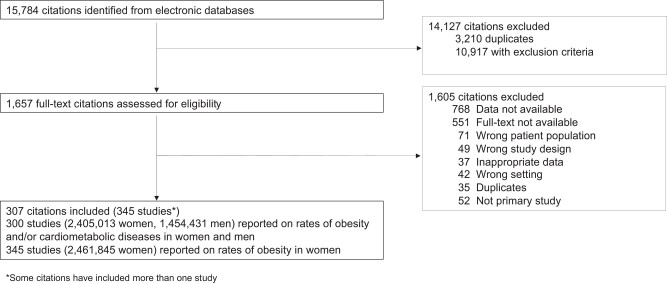
Flow chart of study selection in the systematic review.

We performed a systematic review and meta-analysis using a prospective protocol (https://www.crd.york.ac.uk/prospero/display_record.php? ID=CRD42019132609), PROSPERO CRD42019132609) in line with current recommendations and reported the finding as per PRISMA guidelines (Fig. 1) (13). We searched Embase, Medline, and Cochrane from inception until March 2023. We used MeSH headings, text words, and word variants for “developing country,” such as “low-income” and “middle-income.” We combined them with terms for “obesity,” “comorbidity,” “metabolic disease,” and “women.” There were no language restrictions. Our search strategy complies with the Methodological Expectations of Cochrane Intervention Reviews (MECIR) (14).

Studies were selected for inclusion in 2 stages. First, we screened the title and abstract of all citations for potentially relevant papers and retrieved the full texts of the identified papers for detailed review. Two independent reviewers (T.R. and E.M.) selected the papers against prespecified criteria. Any conflicts were resolved after a discussion with a third reviewer (S.T.).

Studies were included if they assessed the prevalence of obesity by body mass index (BMI) in nonpregnant women in countries classified as low, lower-middle, or upper-middle income by the World Bank regions in the year of the study. We excluded studies including women with cancer, tuberculosis, and HIV infection. Studies that included children, adolescents, animals, and those in high-income countries, as well as letters, case reports, case series, reviews, narrative reviews, case-control studies, and secondary analyses were also excluded. Studies that reported secondary analyses were excluded to prevent bias from including duplicate samples in the meta-analysis. We considered type 2 diabetes, impaired glucose tolerance, dyslipidemia, and nonalcoholic fatty liver disease as metabolic diseases. Hypertension, coronary heart disease, myocardial infarction, and stroke were included as obesity-related cardiovascular diseases. We used the study authors' definitions for obesity and cardiometabolic outcomes for their respective studies (ie, a BMI of ≥27.5 was used in studies in the South Asian community, and ≥30 kg/m^2^ was used in other populations).

### Quality Assessment

Two independent reviewers (T.R. and E.M.) undertook quality assessments using the Newcastle Ottawa Scale (15). The quality assessment tool comprised 3 domains: selection, comparability, and outcomes. A star was given for each subdomain if it was considered adequate. For the selection domain (maximum 5 stars), studies were deemed adequate for representativeness and sample selection if they truly represented the average of the study population (ie, all subjects included or random sampling). Studies that included participants in a nonrandom sampling way but were somewhat representative of the study population were also considered adequate (ie, if the study does not include all participants or randomly sampled but does not appear to be in a selected population). Those with no reports of the sampling strategies were deemed inadequate for representativeness. Studies were also scored adequate for comparability (maximum 2 stars) if the population's age was controlled. The additional score was given if they were controlled for other relevant factors. We considered outcome assessment adequate if the study used a validated measurement tool for the outcome.

### Data Extraction

Data extraction was undertaken by 2 independent reviewers (T.R. and E.M.). We extracted data on the number of women and men with and without obesity and cardiometabolic outcomes such as hypertension, type 2 diabetes, impaired glucose tolerance, dyslipidemia, nonalcoholic fatty liver disease, coronary heart disease, myocardial infarction, and stroke. Where possible, data were extracted for women and men with obesity with outcomes of interest and numbers without obesity. The country’s region and income status were extracted according to the World Bank classification at the time of publication of the study. The setting (urban, rural, or both) and the year of the study (<2000, ≥ 2000) were also assessed.

### Data Analysis

We evaluated the association between sex and the odds of obesity, hypertension, and type 2 diabetes using DerSimonian and Laird random-effects models. We reported the sex-specific associations as summary odds ratios (OR) and 95% CI. We explored whether age (measured in each study as the difference between the mean age of women and men), World Bank region, country's income status (low, lower-middle, or upper-middle), setting (rural, urban, or both), and year of publication (before 2000, or 2000 and after) were significant sources of heterogeneity. Statistical significance for heterogeneity analysis was obtained using likelihood ratio–based omnibus tests in meta-regression analysis. As a measure of statistical heterogeneity, we computed the percentage of variation across studies that is above what is expected by chance (*I²* statistic) (16), and we considered *I^2^* > 50% as substantial heterogeneity. We also estimated the between-study variance (τ²) as a measure of the variability of the underlying true effect distribution.

We summarized the overall prevalence of obesity, hypertension, type 2 diabetes, impaired glucose tolerance, dyslipidemia, nonalcoholic fatty liver disease, coronary heart disease, myocardial infarction, and stroke in adult women in LMICs using multilevel random-effects logistic regression models as implemented in the metapreg Stata user-defined command. We undertook the same heterogeneity analyses as above, performing the same subgroup analyses and fitting similar meta-regression models. In this case, age was considered as the women's mean age in each study. Sensitivity analyses were done by excluding low-quality studies. We refrained from conducting a publication bias assessment due to the substantial heterogeneity in the results, which discourages the use of funnel plot asymmetry tests (17). All analyses were performed using Stata 16 software (StataCorp. 2019. Stata Statistical Software: Release 16. College Station, TX: StataCorp LLC), and the significance level was 5% for all tests.

### Patient and Public Involvement

Patients or members of the public were not involved in our research design, conduct, reporting, or dissemination plans.

### Role of the Funding Source

The study's funders had no role in the study design, data collection, data analysis, data interpretation, or writing of the report. The corresponding author had full access to all the data in the study and took final responsibility for the decision to submit it for publication.

## Results

### Characteristics of the Included Studies

From 15 784 citations, we included 307 citations (63 countries, 345 studies) involving 3 916 276 individuals (2 461 845 women and 1 454 431 men) (Fig. 1). Overall, 55.7% (192/345) of the studies were from Sub-Saharan Africa, 11.6% (40/345) from South Asia, 11.0% (38/345) from Latin America and the Caribbean, 10.4% (36/345) from East Asia and the Pacific, 9.9% (34/345) from the Middle East and North Africa, and 1.4% (5/345) from Europe and Central Asia. Overall, 24.1% (83/345) of the studies were from low-income countries, 41.2% (142/345) were from low-middle, and 34.8% (120/345) were from upper-middle-income countries.

### Quality of the Included Studies

Overall, half (49.8%, 153/307) of all included citations had a low risk of bias. Of the 307 included citations, 46.6% (143/307) were at low risk of selection bias, 44% (135/307) at low risk of comparability bias, and 79.5% (244/307) at low risk of outcome assessment bias. A high risk of selection bias was found in 11.7% (36/307) of the included citations; 42.3% (130/307) presented a high risk of bias for comparability, and 3.6% (11/307) had a high risk of bias for outcome assessment (18).

### Association of Sex With Obesity and Cardiometabolic Diseases

**Figure 2.**
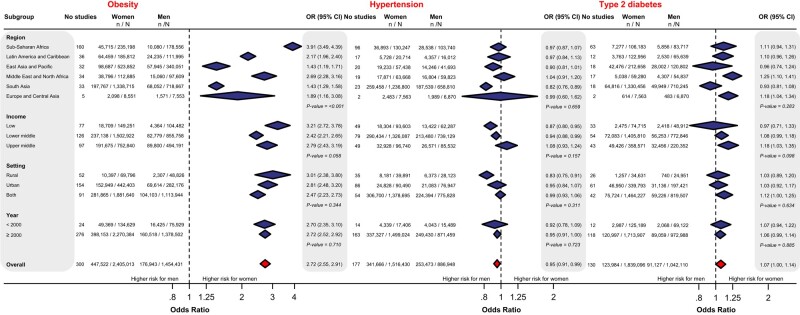
Sex-specific disparities in the odds of obesity, hypertension, and type 2 diabetes in low- and middle-income countries by World Bank region, country's income status, setting, and year.

**Figure 3.**
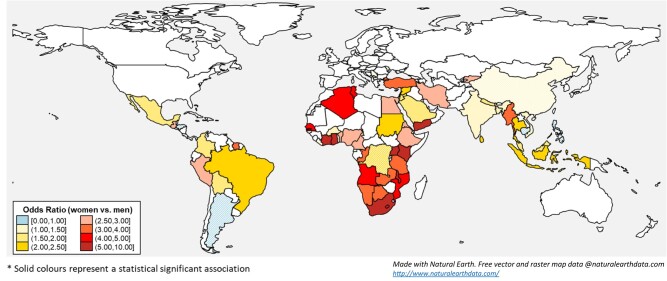
Odds of obesity in women compared with men reported in studies from low- and middle-income countries in the systematic review.

Three hundred studies reported data on obesity in women and men in LMICs. Overall, there was a significant disparity in the odds of obesity between the sexes, with an overall 2.72-fold higher odds of obesity in women than in men (OR 2.72; 95% CI, 2.55-2.91), although the results were highly heterogeneous (*I^2^* = 98.7%, *τ²*=0.29) (Fig. 2) (18). The disparity in the odds of obesity between the sexes was unequal between regions (*P* value < .001). The greatest disparity was observed in studies from Sub-Saharan Africa (OR 3.91; 95% CI, 3.49-4.39; *I^2^* = 94.6%, *τ²*=0.45), followed by the Middle East and North Africa (OR 2.69; 95% CI, 2.28-3.16; *I^2^* = 97.5%, *τ²*=0.20), and Latin America and the Caribbean regions (OR 2.17; 95% CI, 1.96-2.40; *I^2^* = 93.4%, *τ²*=0.06) (Fig. 2) (18). The lowest differences in the odds of obesity between women and men were seen in studies from South Asia (OR 1.43; 95% CI, 1.29-1.59; *I^2^* = 97.7%, *τ²*=0.06) and East Asia and Pacific (OR 1.43; 95% CI, 1.19-1.71; *I^2^* = 98.9%, *τ²*=0.24). Most of the studies from countries in Sub-Saharan Africa reported 3- to 10-fold higher odds of obesity in women than in men (Fig. 3). Similar disparities were observed irrespective of the country´s income status (low, low-middle, upper-middle; *P* value .058), setting (rural, urban; *P* value .344), and year of study (<2000, ≥2000; *P* value .710) (Fig. 2) (18). Age was not associated with the effect of sex on obesity (18). Figure 3 provides estimates of sex-specific disparities in obesity odds in individual LMICs.

Women exhibited a slight decrease in the risk of hypertension (OR 0.95; 95% CI, 0.91-0.99; *I^2^* = 95.7%, *τ²*=0.07) as compared to men while demonstrating a slight increase in the risk of type 2 diabetes (OR 1.07; 95% CI, 1.00-1.14; *I^2^* = 95.2%, *τ²*=0.08) (Fig. 2) (18). We did not find that these effects for hypertension and type 2 diabetes varied by country's income status (*P* values = .157 and .322, respectively), setting (*P* values = .311 and .634, respectively), or over time either (*P* values .723 and .885, respectively) (Fig. 2) (18). Meta-regression analysis found no association between the sex's odds ratio and age imbalances between women and men for obesity and type 2 diabetes. However, this association was significant for hypertension (OR = 1.06, *P* < .001). When women are older than men by a greater difference (x-axis), the impact of sex on hypertension becomes more significant, as indicated by an increasing odds ratio (ie, 6% more per year of age difference) (18). Despite the thorough exploration of sources of heterogeneity, there is still substantial statistical heterogeneity in all subgroups analyzed. Appendixes 10 to 12 of the supplemental material (18) provide estimates of sex-specific disparities in the odds of obesity, hypertension, and type 2 diabetes in individual LMICs.

### Obesity and Cardiometabolic Diseases in Women

**Figure 4.**
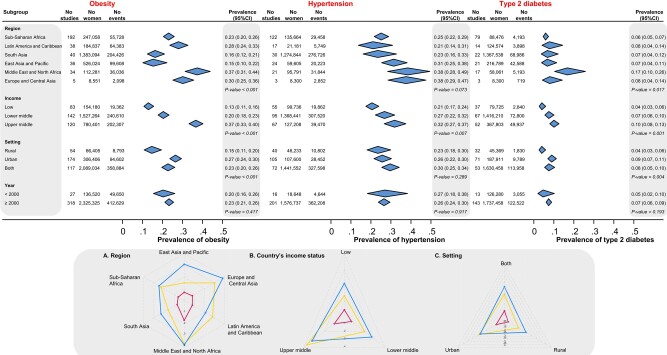
Obesity, hypertension, and type 2 diabetes prevalence in women from low- and middle-income countries reported in studies by World Bank region, country's income status, setting, and year.

Among women in LMICs, the prevalence of obesity was 23.1% (95% CI, 21.1%-25.2%; *I^2^* = 97.6%, *τ²*=1.18; 345 studies), hypertension was 26.5% (95% CI, 23.8%-29.4%; *I^2^* = 97.7%, *τ²*=1.15; 217 studies), and type 2 diabetes was 7.1% (95% CI, 5.9%-8.6%; *I^2^* = 94.4%, *τ²*=1.53; 156 studies) (Table 1). The prevalence of obesity and type 2 diabetes varied among World Bank regions (*P* values <.001 and .017, respectively). The highest obesity prevalence was observed in studies from the Middle East and North Africa (37%; 95% CI, 31%-44%; *I^2^* = 97.7%, *τ²*=0.63), followed by Europe and Central Asia (30%; 95% CI, 25%-36%; *I^2^* = 75.2%, *τ²*=0.06) and Latin America and the Caribbean (28%; 95% CI, 24%-33%; *I^2^* = 96.9%, *τ²*=0.49) (Fig. 4) (18). The highest prevalence of hypertension in women was reported in studies from the Middle East and North Africa (38%; 95% CI, 28%-49%; *I^2^* = 98.5%, *τ²*=1.10) and Europe and Central Asia (38%; 95% CI, 29%-47%; *I^2^* = 96.5%, *τ²*=0.13), followed by East Asia and Pacific (31%; 95% CI, 25%-38%; *I^2^* = 98.6%, *τ²*=0.54) (Fig. 4) (18). Type 2 diabetes prevalence in women was highest in studies from the Middle East and North Africa (17%; 95% CI, 10%-26%; *I^2^* = 98.6%, *τ²*=1.32), followed by Europe and Central Asia (8%; 95% CI, 4%-14%; *I^2^* = 95.6%, *τ²*=0.32), and Latin America and the Caribbean (8%; 95% CI, 4%-14%; *I^2^* = 94.2%, *τ²*=1.65) (Fig. 4) (18). Table 1 provides the prevalence of other cardiometabolic conditions in women, such as impaired glucose intolerance, dyslipidemia, and nonalcoholic fatty liver disease. The reported prevalence of coronary heart disease, myocardial infarction, stroke, and obstructive sleep apnea syndrome was 5.3%, 5.1%, 2.8%, and 22.1%, respectively (Table 1).

**Table 1.** Reported prevalence of obesity and cardiometabolic diseases in women in low- and middle-income countries

Risk factor	Number of studies	Number of women	Number of women with the event	Prevalence (95% CI)	*I^2^* (%)	*T^2^*
**Obesity**	345	2 461 845	462 279	0.231 (0.211, 0.252)	97.6	1.18
**Hypertension**	217	1 595 385	366 852	0.265 (0.238, 0.294)	97.7	1.15
**Type 2 diabetes**	156	1 863 738	125 577	0.071 (0.059, 0.086)	94.4	1.53
**Impaired glucose tolerance**	51	74 693	6654	0.097 (0.070, 0.132)	94.9	1.59
**Dyslipidemia**	31	56 531	35 193	0.373 (0.268, 0.492)	97.6	1.88
**Nonalcoholic fatty liver disease**	3	11 876	1660	0.190 (0.088, 0.365)	99.5	0.62
**Coronary heart disease**	20	148 170	3302	0.053 (0.028, 0.098)	90.3	2.21
**Myocardial infarction**	6	8128	259	0.051 (0.030, 0.083)	77.6	0.37
**Stroke**	14	121 265	821	0.028 (0.012, 0.065)	88.7	2.70
**Obstructive sleep apnea syndrome**	4	1033	239	0.221 (0.182, 0.267)	40.4	0.03

*T* ^2^ is an estimation of the between-studies variance. It can be interpreted as the square of the SD of the underlying distribution of true effects.

*I* ^2^ represents the proportion of total variation across studies that is above the variation expected by chance.

**Figure 5.**
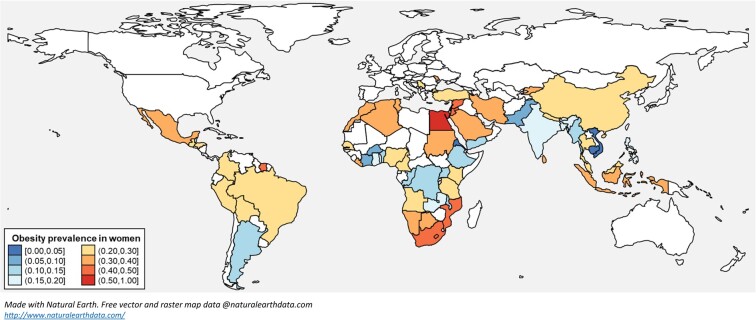
Obesity prevalence in women from various low- and middle-income countries in the studies included in the systematic review.

The prevalence of obesity, hypertension, and type 2 diabetes in women varied significantly by the country's income status (*P* values: < .001, .007, and .001, respectively), with the highest prevalence in upper-middle-income countries and the lowest in low-income countries. Obesity and type 2 diabetes prevalence in women was highest in urban settings, with estimates of 27% (95% CI, 24%-30%; *I^2^* = 97.2%, *τ²*=1.00) and 9% (95% CI, 7%-11%; *I^2^* = 93.9%, *τ²*=1.22), respectively (Fig. 4). Figure 5 and Appendix 20 (18) provide obesity prevalence in women in studies from individual LMICs. The meta-regression analysis has shown that as women’s age increased, there was a significant rise in the prevalence of obesity, hypertension, and type 2 diabetes. Specifically, for every 10 years of age, the odds of obesity increased by 51% (OR 1.04 per year, *P* < .001), hypertension by 98% (OR 1.07, *P* < .001), and type 2 diabetes by 93% (OR 1.07, *P* < .001) (18). Supplemental data appendixes 20 to 23 (18) provide the prevalence of obesity, hypertension, and type 2 diabetes in individual LMICs. The prevalence of obesity, hypertension, and type 2 diabetes remained similar when a sensitivity analysis was performed, excluding low-quality studies (18).

### Association Between Obesity and Cardiometabolic Diseases in Women

In women with obesity in LMICs, there was an overall 2-fold increase in the odds of hypertension (OR 2.41; 95% CI, 1.89-3.08; 21 studies) and type 2 diabetes (OR 2.65; 95% CI, 1.76-3.98-3.74; 16 studies), and 3-fold increase for impaired glucose tolerance (OR 3.06; 95% CI, 1.84-5.11) (Table 2) (18). Sensitivity analysis limited to studies adjusted for at least one variable (such as age and education level) found similar results (Table 2). Likewise, the estimates did not significantly change when the analysis was restricted to studies with low risk of bias (Table 2).

**Table 2.** Odds of cardiometabolic diseases in women with vs without obesity in low- and middle-income countries

Cardiometabolic Condition	Studies includedin analysis	No. of studies	No. of women with obesity	No. of women without obesity	Odds ratio (95% CI)	*I^2^* (%)	*T^2^*
**Hypertension**	Adjusted studies	9	11 413	28 789	2.38 (1.82, 3.12)	93.2	0.115
	All studies	21	26 167	68 849	2.41 (1.89, 3.08)	97.5	0.270
	High quality studies	17	23 124	61 740	2.42 (1.81, 3.24)	97.9	0.319
**Type 2 diabetes** *^a^*	Adjusted studies	8	19 915	103 000	2.66 (2.06, 3.42)	68.5	0.071
	All studies	16	31 372	146 453	2.65 (1.76, 3.98)	96.3	0.574
	High quality studies	9	27 776	127 020	2.56 (1.58, 4.16)	96.2	0.462
**Impaired glucose tolerance** *^a^*	All studies	4	374	2731	3.06 (1.84, 5.11)	46.8	0.121
	High quality studies	1	282	306	4.59 (3.14, 6.72)	··	··
**Dyslipidemia**	Adjusted studies	1	1704	2655	1.41 (1.20, 1.66)	··	··
	All studies	3	1816	2780	1.04 (0.60, 1.80)	56.7	0.135
	High quality studies	1	1704	2655	1.41 (1.20, 1.66)	··	··
**Low HDL-cholesterol** *^b^*	All studies	1	46	89	1.96 (0.61, 6.34)	··	··
**Nonalcoholic fatty liver disease** *^b^*	All studies	1	247	8074	25.09 (19.10, 32.97)	··	··
**Coronary heart disease**	Adjusted studies	1	9623	19 187	1.60 (1.40, 1.83)	··	··
	All studies	1	9623	19 187	1.60 (1.40, 1.83)	··	··
	High quality studies	1	9623	19 187	1.60 (1.40, 1.83)	··	··
**Myocardial infarction** *^b^*	All studies	1	46	89	0.97 (0.17, 5.48)	··	··
**Obstructive sleep apnea syndrome** *^a^*	All studies	1	226	350	2.03 (1.40, 2.97)	··	··
	High quality studies	1	226	350	2.03 (1.40, 2.97)	··	··

*T* ^2^ is an estimation of the between-studies variance. It can be interpreted as the square of the SD of the underlying distribution of true effects.

*I* ^2^ represents the proportion of total variation across studies that is above the variation expected by chance.

^*a*^Unadjusted studies.

^*b*^Unadjusted studies and no high quality (low risk of bias) study.

## Discussion

In low- and middle-income countries, the odds of obesity are approximately 3 times higher for women than men, irrespective of the country's income status (low, lower-middle or upper-middle), study setting (rural or urban) or year of the study. The Sub-Saharan region has the greatest disparity between women and men in the reported odds of obesity. Individual studies reported that the odds of obesity in women are 3 to 10 times higher than in men. Although with small effect, slight differences between the sexes are seen in the odds of reported hypertension and type 2 diabetes, with women having a decreased risk of hypertension but a slight increase in the risk of type 2 diabetes than men. Women in the Middle East and North Africa region, upper-middle-income countries, and urban settings have the highest obesity and type 2 diabetes prevalence. Obesity is associated with 2- to 3-fold odds of hypertension and type 2 diabetes in women in LMICs.

To our knowledge, ours is the first meta-analysis to quantify the sex-related disparities in LMICs for obesity, hypertension, and type 2 diabetes. We identified the World Bank regions, the country's income status, and settings where women are disproportionately affected. Additionally, we provided a detailed mapping of obesity and cardiometabolic disease burden in women in LMICs. Our systematic review was performed using a comprehensive search without language restrictions. We tested the robustness of our findings by undertaking a sensitivity analysis that excluded high risk of bias studies. Our results were broadly similar when the analysis was limited to studies adjusted for at least one variable (such as age) and those with a low risk of bias. We adjusted our analysis for the age of the participants to consider variations in risks with age. We ensured that the findings accurately reflected the country's income status when the study was conducted by considering the World Bank classification at the year the study was conducted.

Our study was limited by the heterogeneities in the assessment of cardiometabolic diseases. Many studies included participants with self-reported conditions, which could bias our estimates. However, our estimates of obesity, hypertension, and type 2 diabetes prevalence in women are similar to those reported in nationally representative observational studies on LMICs (8, 19, 20). We have observed some small studies providing unrealistic estimates of the association between obesity and hypertension that could have been affected by sparse-data bias (21). We only reported outcomes such as obesity, hypertension, and type 2 diabetes and not cardiovascular events such as myocardial infarction, stroke, and death due to the paucity of reported data. Data were available for only about half of all the LMICs worldwide. Hence, some regions and countries were represented less than others, particularly in North Africa and East Asia. While we only included cohort studies, these were not always nationally representative estimates.

Nevertheless, about half of all included studies had a low risk of bias for the representativeness of the population. We did not include pregnant women, as we felt it was inappropriate to combine pregnancy-related data with data from nonpregnant women or to compare outcomes with men. Finally, we could not stratify the risk of obesity according to sex by age or by individual socioeconomic status. Meta-regression analysis showed that sex-specific obesity odds persisted independent of the age difference between women and men. Additionally, meta-regression showed that the odds of obesity, hypertension, and type 2 diabetes are positively associated with women's age.

The high burden of obesity in women in LMICs can be explained by underlying biological, sociocultural, and socioeconomic factors. Women-specific factors such as gestational weight gain, parity, polycystic ovary syndrome, and menopause are well-known to be associated with an increased risk of obesity (3, 22-25). Additionally, early life conditions may have permanent sex-specific effects on appetite regulation, eating behaviors, and body weight gain patterns (26-28). Lastly, obesity risk seems to be positively and significantly associated with childhood deprivation in women but not men (29). Women are also more likely to be influenced than men by other factors predisposing them to obesity, such as poor dietary habits, sedentary lifestyles, and price inflation (30). Moreover, sociocultural aspects such as the perception of ideal body size and beliefs surrounding the acceptability of physical activities are related to the high burden of obesity in women in the Middle East and North Africa and Sub-Saharan Africa regions. Beliefs that larger body types indicate high socioeconomic status and fertility associate obesity in women as a sign of “wealth and health” (31-33). Finally, LMICs' rapid economic growth and urbanization levels result in shifts in dietary and physical activity patterns that contribute to a rapid increase in obesity prevalence combined with a decline in undernutrition (34, 35).

We observed a slight difference in hypertension and type 2 diabetes prevalence between women and men. A Noncommunicable Diseases Risk Factor Collaboration study arm showed a significant rise in diabetes prevalence in LMICs from 1980 to 2014 but, in contrast with our results, observed a shift in diabetes burden from women toward men (36). Different findings in the global and regional sex-specific prevalence of type 2 diabetes in LMICs could have been influenced by variations in the reported prevalence of diabetes and selective diagnosis. Other reasons for different results might include variabilities in the diabetes risk according to different BMI categories by region and sex (20).

We could not establish causal relationships between obesity, type 2 diabetes, and hypertension, as our study design did not control the temporal relationship between these factors. Still, not surprisingly, some regions that reported high obesity prevalence in women also showed a high prevalence of hypertension and type 2 diabetes, such as the Middle East and North Africa region. However, the magnitude of the burden of type 2 diabetes in women in LMICs did not always follow the burden of obesity and hypertension. This can be explained by variabilities in the risk of type 2 diabetes across different BMI categories by region and sex, as demonstrated in a cross-sectional study of nationally representative data on the association between BMI and diabetes in LMICs (20). Potential reasons for divergent type 2 diabetes prevalence may include underdiagnosis, considering that anthropometry and blood pressure measurements are low-cost and more easily assessed to diagnose obesity and hypertension than the blood tests required to ascertain diabetes. Despite having low levels of obesity, women in East Asia and the Pacific presented with the third-highest prevalence of hypertension. This is similar to the 30% observed in population-based studies from the region and suggests contributors to hypertension other than obesity, such as genetic predisposition (19).

The association between obesity and an increased risk of cardiometabolic diseases such as hypertension and type 2 diabetes in women is consistent with the findings reported in studies from high-income countries, highlighting common pathophysiological pathways (12, 20). The positive association of women's odds of obesity, hypertension, and type 2 diabetes with women's age relates to the female-specific postmenopausal changes that predispose women to changes that affect overall energy expenditure and basal metabolic rates, such as abdominal obesity and muscle loss, and contribute to weight gain in women in midlife (37, 38).

Our findings call for urgent sex-specific and region-stratified actions targeting obesity awareness, prevention, treatment, and control in women in LMICs. Interventions require a woman-centred approach considering the cultural, social, and behavioural barriers and facilitators uniquely faced by women in following the recommended diet-based and physical activity interventions and appropriate pharmacotherapy. Public health messages should address underlying cultural misconceptions where obesity is perceived as a sign of “wealth and health.” Early commencement of preventative interventions and regular follow-ups could also help reduce the burden of cardiovascular diseases in women. Targeted efforts are needed for early diagnosis and care of women-specific conditions such as excess weight gain in pregnancy, gestational diabetes, and polycystic ovary syndrome, predisposing women to long-term cardiometabolic diseases. Further research is needed to investigate how cultural aspects shape body weight perceptions in women and how best to conciliate those to community-level interventions.

## Data Availability

Some or all datasets generated during and/or analyzed during the current study are not publicly available but are available from the corresponding author upon reasonable request.

## Abbreviations

BMI: body mass index
LMIC: low- and middle-income country
OR: odds ratio
